# Novel Therapeutic Potentials of Taxifolin for Obesity-Induced Hepatic Steatosis, Fibrogenesis, and Tumorigenesis

**DOI:** 10.3390/nu15020350

**Published:** 2023-01-10

**Authors:** Takayuki Inoue, Bin Fu, Miwako Nishio, Miyako Tanaka, Hisashi Kato, Masashi Tanaka, Michiko Itoh, Hajime Yamakage, Kozue Ochi, Ayaka Ito, Yukihiro Shiraki, Satoshi Saito, Masafumi Ihara, Hideo Nishimura, Atsuhiko Kawamoto, Shian Inoue, Kumiko Saeki, Atsushi Enomoto, Takayoshi Suganami, Noriko Satoh-Asahara

**Affiliations:** 1 Department of Endocrinology, Metabolism and Hypertension Research, Clinical Research Institute, National Hospital Organization Kyoto Medical Center, Kyoto 612-8555, Japan; 2 Department of Molecular Medicine and Metabolism, Research Institute of Environmental Medicine, Nagoya University, Nagoya 464-8601, Japan; 3 Department of Immunometabolism, Nagoya University Graduate School of Medicine, Nagoya 464-8601, Japan; 4 Department of Laboratory Molecular Genetics of Hematology, Graduate School of Medical and Dental University, Tokyo Medical and Dental University, Tokyo 113-8510, Japan; 5 Institute of Nano-Life-Systems, Institutes of Innovation for Future Society, Nagoya University, Nagoya 464-8601, Japan; 6 Department of Physical Therapy, Health Science University, Yamanashi 401-0380, Japan; 7 Institute for Advanced Research, Nagoya University, Nagoya 464-8601, Japan; 8 Department of Pathology, Nagoya University Graduate School of Medicine, Nagoya 466-8550, Japan; 9 Department of Neurology, National Cerebral and Cardiovascular Center, Osaka 564-8565, Japan; 10 Translational Research Center for Medical Innovation, Foundation for Biomedical Research and Innovation at Kobe, Kobe 650-0047, Japan; 11 Center for One Medicine Innovative Translational Research, Gifu University Institute for Advanced Study, Gifu 501-11193, Japan; 12 Department of Metabolic Syndrome and Nutritional Science, Research Institute of Environmental Medicine, Nagoya University, Nagoya 466-8550, Japan

**Keywords:** Taxifolin, obesity, antioxidant, nonalcoholic steatohepatitis (NASH), inflammation, brown adipocytes, fibroblast growth factor-21

## Abstract

The molecular pathogenesis of nonalcoholic steatohepatitis (NASH) includes a complex interaction of metabolic stress and inflammatory stimuli. Considering the therapeutic goals of NASH, it is important to determine whether the treatment can prevent the progression from NASH to hepatocellular carcinoma. Taxifolin, also known as dihydroquercetin, is a natural bioactive flavonoid with antioxidant and anti-inflammatory properties commonly found in various foods and health supplement products. In this study, we demonstrated that Taxifolin treatment markedly prevented the development of hepatic steatosis, chronic inflammation, and liver fibrosis in a murine model of NASH. Its mechanisms include a direct action on hepatocytes to inhibit lipid accumulation. Taxifolin also increased brown adipose tissue activity and suppressed body weight gain through at least two distinct pathways: direct action on brown adipocytes and indirect action via fibroblast growth factor 21 production in the liver. Notably, the Taxifolin treatment after NASH development could effectively prevent the development of liver tumors. Collectively, this study provides evidence that Taxifolin shows pleiotropic effects for the treatment of the NASH continuum. Our data also provide insight into the novel mechanisms of action of Taxifolin, which has been widely used as a health supplement with high safety.

## 1. Introduction

Increasing attention has been paid to nonalcoholic steatohepatitis (NASH), a hepatic phenotype of the metabolic syndrome, because NASH progressively develops into cirrhosis and hepatocellular carcinoma in the long term. To date, numerous clinical trials for NASH have been conducted globally; however, there are no approved therapeutic strategies for NASH [1]. As the “multiple parallel hits” hypothesis suggests, the molecular pathogenesis of NASH includes the complex interaction of metabolic abnormalities, such as insulin resistance and lipid accumulation and inflammatory stimuli, including endotoxins and proinflammatory cytokines [2]. Therefore, chemical compounds possessing pleiotropic effects may be applicable for treating NASH. Indeed, combinations of chemical compounds for distinct molecular targets have been under clinical trials.

Considering the therapeutic goals of NASH, it is important to determine whether the treatment can prevent its progression to hepatocellular carcinoma, in addition to ameliorating hepatic steatosis, metabolic derangements, and liver fibrosis. A bottleneck of NASH research is the limited experimental NASH models that exhibit human NASH-like liver phenotypes. In this respect, we have shown that genetically obese melanocortin 4 receptor (Mc4r)-deficient mice on a high-fat diet progressively develop hepatic steatosis, NASH, and multiple liver tumors [3]. Using this unique experimental model, we assessed the effects of several chemical compounds such as sodium-glucose cotransporter-2 inhibitors on the development of NASH and subsequent liver tumors [4,5,6].

Taxifolin, also known as dihydroquercetin, is a natural bioactive flavonoid commonly contained in various foods, such as green tea, fruits, and several herbs, such as milk thistle [7]. It is also included in health supplements including silymarin [7]. Based on its antioxidant and anti-inflammatory properties, accumulating evidence has indicated that Taxifolin potently ameliorates various disease models including cardiovascular diseases [8,9,10]. Moreover, we have demonstrated the therapeutic potential of Taxifolin for amyloid-β oligomer formation and cognitive dysfunction in a murine model of Alzheimer’s disease [11,12]. Taxifolin also mitigates the development of obesity and glucose intolerance in certain experimental models [13,14,15], although the molecular mechanisms of action are currently unknown. These observations indicate the pleiotropic effects of Taxifolin. In addition, several studies have pointed to the protective effects of Taxifolin with the dose of 20–200 mg/kg/day by oral gavage daily for 7 to 28 days on chemically induced liver injury in mice [16,17,18]. In addition, suppressive effects of Taxifolin have been reported in studies related to the acute alcohol–induced liver injury in mice [19]. However, the therapeutic efficacy of Taxifolin on NASH and subsequent liver tumors remains to be elucidated.

In this study, we demonstrated that Taxifolin treatment markedly prevented the development of lipid accumulation, chronic inflammation, and fibrosis of the liver in a murine NASH model. Its mechanisms include suppressing body weight gain, at least partly, through increasing brown adipose tissue activity. Taxifolin may also directly act on hepatocytes to inhibit lipid accumulation. Moreover, Taxifolin treatment after NASH development could effectively prevent its progression to liver tumors. Collectively, this study provides evidence that Taxifolin shows pleiotropic effects for the treatment of obesity-induced hepatic steatosis, fibrogenesis, and tumorigenesis.

## 2. Materials and Methods

### 2.1. Materials

All reagents and materials were obtained from Sigma-Aldrich (St. Louis, MO, USA), Cell Signaling Technology (CST, Beverly, MA, USA), or Nacalai Tesque (Kyoto, Japan), unless otherwise noted.

### 2.2. Animals

The C57BL/6J mice were obtained from CLEA Japan. Fibroblast growth factor 21 (Fgf21)-deficient mice and Mc4r-deficient mice on the C57BL/6J background were kindly gifted by Nobuyuki Itoh (Kyoto University, Kyoto, Japan) and Joel K. Elmquist (University of Texas Southwestern Medical Center), respectively. The animals were housed in a temperature-, humidity-, and light-controlled animal room (12 h light and 12 h dark cycle) and allowed free access to food and water. All animal experiments were carried out according to the ARRIVE guidelines.

### 2.3. Diet-Induced Obesity Model

The eight-week-old male C57BL/6J mice were fed a standard diet (SD) or a high-fat diet (HD) (HFD-60; 506 kcal/100 g, 60% energy as fat; Oriental Yeast, Tokyo, Japan) with or without Taxifolin (0.05% for the low-dose group (TX-L) and 3% for the high-dose group (TX-H); Ametis JSC, Blagoveshchensk, Russia). Twelve weeks after the start of the experiment, an intraperitoneal glucose tolerance test (IPGTT; 1.0 g/kg body weight) was performed under overnight fasting conditions. For the experiments using Fgf21-deficient mice, the mice were fed an SD or a TX-H for 6 weeks. The rectal temperature was evaluated with a thermometer (Physitemp BAT7001H, Fisher scientific, Clifton, NJ) 8 weeks after the start of the experiment. The mice were sacrificed after overnight fasting under intraperitoneal pentobarbital anesthesia (30 mg/kg) at the end of each experiment.

### 2.4. NASH and Liver Tumor Models

To examine the preventive effects of Taxifolin in a NASH model, 8 week old male Mc4r-deficient mice were fed a Western diet (WD) (D12079B; 468 kcal/100 g, 41% energy as fat, 34.0% sucrose, 0.21% cholesterol; Research Diets, New Brunswick, NJ, USA) with or without 3% Taxifolin for up to 20 weeks. As a control, wild-type mice were fed an SD. To examine the therapeutic effects, Mc4r-deficient mice were fed a WD for 16 weeks, and then the mice were treated with or without 3% Taxifolin for an additional 8 weeks. For evaluating the effects on hepatocellular carcinoma development, the Mc4r-deficient mice were fed a WD for 20 weeks, and then the mice were treated with or without 3% Taxifolin for an additional 30 weeks. The mice were sacrificed, when fed ad libitum, under intraperitoneal pentobarbital anesthesia (30 mg/kg) at the end of each experiment.

### 2.5. Blood Analysis

The concentrations of blood glucose, serum alanine aminotransferase (ALT), aspartate aminotransferase (AST), total cholesterol (TC), triglyceride (TG), and nonesterified fatty acid (NEFA) were measured as described previously [11,20]. The serum concentrations of insulin and FGF21 were measured by means of commercially available ELISA kits (Morinaga Ultra Sensitive Mouse Insulin ELISA kit (Morinaga Institute of Biological Science, Kanagawa, Japan) and Mouse FGF21 ELISA kit (R&D Systems, MN, USA), respectively). The homeostasis model assessment of insulin resistance (HOMA-IR) was calculated as (fasting serum glucose × fasting serum insulin (mg/dL × ng/mL)) to assess the insulin resistance.

### 2.6. Lipid Contents and Hydroxyproline Levels of the Liver

The hepatic total lipids were extracted with ice-cold 2:1 (vol/vol) chloroform/methanol, and the triglyceride and cholesterol contents were measured by commercially available kits (FUJIFILM Wako Pure Chemical, Osaka, Japan). The hepatic hydroxyproline levels were determined as described previously [5].

### 2.7. Serum and Hepatic Malondialdehyde Contents

The serum and hepatic malondialdehyde (MDA) contents were measured using a Colorimetric TBARS Microplate Assay kit (Oxford Biomedical Research, Upper Heyford, UK) according to the manufacturer’s instructions.

### 2.8. Quantitative Real-Time PCR

Quantitative real-time PCR was conducted as previously described [21]. In brief, the total RNA was extracted from cultured cells or tissues using RNeasy Mini kit (QIAGEN, Germantown, MD, USA), and real-time PCR amplification was performed with the SYBR GREEN detection protocol in a thermal cycler (StepOne Plus; Thermo Fisher Scientific, Waltham, MA, USA). The primers used in this study are listed in Supplementary Materials Tables S1 and S2. As internal controls, 18s, 36B4, or GAPDH was used, and the data were normalized by the comparative cycle threshold method.

### 2.9. Western Blotting

Western blotting analysis was performed as described with minor modifications [22]. Homogenate from the liver was prepared with a RIPA lysis buffer (150 mM NaCl, 1% NP-40, 0.5% sodium deoxycholate, 50 mM Tri-HCl (pH 7.4) supplemented with HaltTM Protease and Phosphatase Inhibitor Cocktail (Thermo Scientific, Tokyo, Japan). The same concentration of protein (20–40 µg per each sample) was resolved by SDS-polyacrylamide gel electrophoresis and then transferred to PVDF membranes. The membrane was blocked with a blocking solution (Nacalai Tesque), followed by incubation with the following primary antibodies: anti-FAS (diluted 1:1000; #3180; Cell Signaling Technology, CST), anti-ACC (diluted 1:1000; #3676; CST), anti-SCD-1 (diluted 1:1000; #2794; CST), anti-TNFα (diluted 1:800; #11948; CST), and anti-β-actin (diluted 1:3000; #4980; CST). After washing, each band was incubated with an HRP-conjugated anti-rabbit IgG secondary antibody (#7074; CST) and detected with the ECL Prime Western Blotting Detection System (GE Healthcare, Uppsala, Sweden). We captured each band images using the ChemiDoc XRS Plus imaging system (Bio-Rad, Hercules, CA, USA) and quantified the protein levels by analyzing the band intensities using ImageJ (NIH, Bethesda, MD, USA).

### 2.10. Histological Analysis

The histological analysis was performed as described [4,5,23]. Four-micromillimeter-thick paraffin-embedded liver sections were stained with hematoxylin and eosin and Sirius red. Type III collagen and F4/80-positive macrophages were immunohistochemically detected using anti-type III collagen (1330-01, SouthernBiotech, Birmingham, AL, USA) and anti-F4/80 (MCA497GA, Bio-Rad Laboratories, Hercules, CA, USA) antibodies, respectively [23]. Liver fibrosis was measured as positive areas for Sirius red or type III collagen using BZ-X710 (KEYENCE, Osaka, Japan). F4/80 immunostaining was used to detect crown-like structures (CLS), and the number of CLS was counted in the whole area of each section. Following the NASH clinical research network scoring system, the scores for steatosis, inflammation, and hepatocyte ballooning were assessed. The stages of fibrosis were determined with Sirius red staining. For the assessment of tumor development, lumps were analyzed in the liver, in which the lumps less than 1 mm and larger than 1 mm were considered as foci and tumors, respectively (Supplementary Materials Figure S1). The histological evaluation for the presence of histologically malignant areas (i.e., carcinoma-like lesions) and microscopic dysplastic nodules were performed independently by two board-certified pathologists (Y. S. and A. E.), according to the guidelines of the 5th edition of the WHO *Classification of Tumors* of the digestive system. The areas of individual macroscopic tumors, HCC-like lesions, and dysplastic nodules were measured using ImageJ software (version 1.51j8), followed by the quantification of the percentage of HCC-like lesions in the entire macroscopic tumors with diameters more than 2 mm. The microscopic dysplastic nodules were defined as areas with diameters between 0.5 and 2 mm that are composed of atypical hepatocytes with a clonal appearance.

### 2.11. Experiments Using HepG2

The human hepatocellular carcinoma cell line, HepG2, was purchased from the American Type Culture Collection (Manassas, VA). HepG2 was cultured in high-glucose Dulbecco’s modified Eagle’s medium (DMEM) supplemented with 10% fetal bovine serum (BSA), 100 U/mL penicillin, and 100 μg/mL streptomycin and incubated in 5% CO_2_ at 37 °C. The effects of Taxifolin on cell viability were evaluated using the 3-(4,5-dimethylthiazol-2-yl)-2,5-diphenyl tetrazolium bromide (MTT) assay according to the manufacturer’s instructions (Nacalai Tesque). The administration of palmitic acid (PA) was conducted as previously described with minor modifications. [24]. The PA (Sigma-Aldrich) was solubilized in ethanol until the PA particles were completely dissolved. Then, the PA was combined with fatty-acid-free BSA solution at a volume ratio of 10:1 (BSA sol.: PA sol.) immediately and with sufficient mixing at 37 °C. A control solution containing ethanol and BSA was prepared similarly. The HepG2 cells were treated with PA at 400 µM for 24 h. The lipid accumulation of the HepG2 cells were also assessed by Oil Red O staining. Briefly, the HepG2 cells were with PBS, fixed in formalin (10%) for 1 h, stained with Oil Red O solution for 1 h, and washed with distilled water. To quantitate the lipid contents, Oil Red O was extracted from each well with isopropanol and read spectophotometrically at 540 nm.

### 2.12. Experiments Using Human iPS Cell-Derived Brown Adipocytes

The human iPS cell line (hiPSCs) was established from human umbilical vein endothelial cells by introducing Yamanaka factors (Oct3/4, Sox2, Klf4, and c-Myc) using CytoTune-iPS ver.1.0 (ID Pharma, Ibaraki, Japan). The hiPSCs were maintained by the feeder-free system (StemFit AK02N, Ajinomoto Healthy Supply, Tokyo, Japan) on 60 mm diameter plates precoated with iMatrix (10 µL/60 mm plate) (Nippi, Tokyo, Japan). The media were changed every other day. Once the cell density reached a 70–80% confluency, the hiPSCs were treated by a treatment with 1 mL TrypLE Express (Thermo Fisher Scientific) for 5–10 min at 37 °C and collected by gentle pipetting with a 100 µL tip. After washing with PBS, the hiPSCs were seeded onto new 60 mm diameter precoated plates (0.5–1 × 105 cells/plate) using 4 mL StemFit AK02N supplemented with 40 µL RevitaCell supplement (Thermo Fisher Scientific), ROCK inhibitor. The cell proliferation rate was 2.0 logs in 5–7 days. To differentiate the hiPSCs into brown adipocytes, the hiPSCs were harvested, dissociated into single cells by TrypLE Express treatment, and suspended in differentiation medium with RevitaCell supplement. The cells were cultured at 37 °C in a CO_2_ incubator (5% CO_2_) for 6–8 days. Half of the differentiation media were refreshed every other day. Then, hiPSC-derived brown adipocytes were treated with 100 µM Taxifolin for 48 h.

### 2.13. Statistical Analysis

The data are expressed as the mean ± SEM. The statistical analysis was conducted using one-way ANOVA followed by the Tukey–Kramer test. The comparisons of the body weight and serum glucose concentrations during the IPGTT were performed using a two-way factorial ANOVA with repeated measurement followed by the Tukey–Kramer test. A *p*-value < 0.05 was considered statistically significant. The analyses were performed with GraphPad Prism version 9 (GraphPad Software, San Diego, CA, USA).

## 3. Results

### 3.1. Preventive Effects of Taxifolin on Body Weight Gain, Metabolic Derangements, and Hepatic Steatosis in Diet-Induced Obese Mice

**Figure 1:**
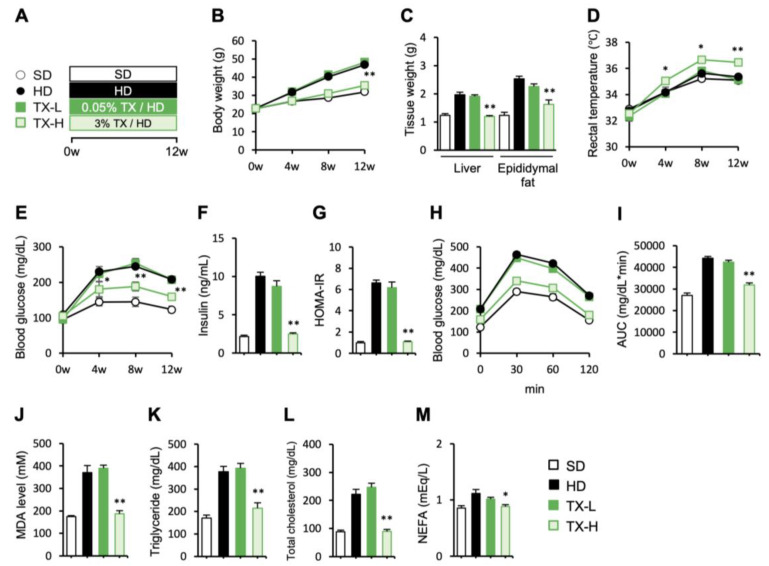
The preventive effects of Taxifolin on obesity and metabolic derangements in diet-induced obese mice. (**A**) Experimental protocol: male C57BL/6J mice were divided into the following 4 groups—SD group with a standard diet, HD group with a high-fat diet, TX-L group with a high-fat diet containing 0.05% (wt/wt) of Taxifolin, and TX-H group with a high-fat diet containing 3% (wt/wt) of Taxifolin. *n* = 6 in each group. (**B**) Growth curve; (**C**) tissue weights; (**D**) rectal temperature; (**E**–**G**) blood glucose levels (**E**), serum insulin concentrations (**F**), and homeostasis model assessment of insulin resistance (HOMA-IR) under fasting conditions (**G**); (**H**,**I**) intraperitoneal glucose tolerance test (injection of 1.0 g/kg of glucose) after 12 weeks of high-fat diet feeding: (**H**) blood glucose levels; (**I**) area under the curve (AUC) values for the blood glucose concentrations during the glucose tolerance test; (**J**–**M**) serum concentrations of malondialdehyde (MDA), triglyceride, total cholesterol, and nonesterified fatty acid (NEFA). Values are presented as the means ± SEM; significant differences: * *p* < 0.05 and ** *p* < 0.01 vs. HD.

To test the therapeutic potentials of Taxifolin in obesity, different doses of Taxifolin were orally administered to male C57BL6/J mice fed an HD for 12 weeks (Figure 1A). Taxifolin treatment dose-dependently suppressed the increase in body weight and liver and epididymal fat weights (Figure 1B,C). The mice fed an HD containing high-dose Taxifolin (TX-H) had a significantly increased rectal temperature relative to those fed a control HD (Figure 1D), although there was no significant difference in the food intake between the treatments. For the metabolic parameters, the blood glucose concentrations under fasted conditions were significantly lower in the TX-H group than in the HD group (Figure 1E). The serum insulin concentrations and HOMA-IR were also suppressed in the TX-H group (Figure 1F,G). The intraperitoneal glucose tolerance test confirmed the ameliorated glucose metabolism by Taxifolin treatment (Figure 1H,I). As it is known as an antioxidant, the Taxifolin treatment significantly reduced the serum MDA concentrations in the diet-induced obese mice (Figure 1J). In addition, the serum levels of triglyceride, total cholesterol, and NEFA were significantly lower in the TX-H group than in the HD group (Figure 1K–M).

**Figure 2:**
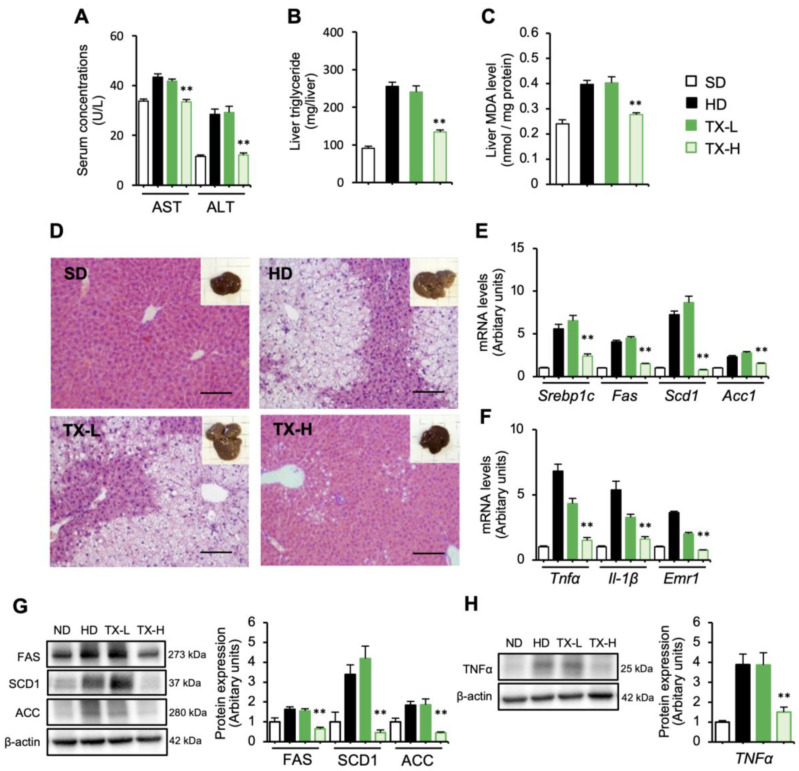
Preventive effects of Taxifolin on hepatic steatosis in diet-induced obese mice. White square: SD; black square: HD; dark-green square: TX-L; light-green square: TX-H. *n* = 6 in each group. (**A**) Serum concentrations of AST and ALT after 12 weeks of HD feeding; (**B**,**C**) hepatic triglyceride and MDA contents; (**D**) hematoxylin and eosin (HE) staining of the liver. Insets: gross appearance of the livers. Scale bars: 100 µm. (**E**,**F**) Expression levels of genes related to lipogenesis (*Srebp1c*, *Fas*, *Scd1*, and *Acc1*) and inflammation (*Tnfα*, *Il1b*, and *Emr1* (F4/80)) in the liver; (**G**,**H**) immunoblot analysis of the protein expression levels related to lipogenesis (FAS, SCD-1, and ACC) and inflammation (TNFα) in the liver. β-actin was used as a loading control. Values are presented as the means ± SEM; *n* = 6; significant differences: ** *p* < 0.01 vs. HD.

We further examined the effects of Taxifolin on hepatic steatosis and found that serum concentrations of AST and ALT, along with the hepatic contents of triglyceride and MDA, were dose-dependently reduced by Taxifolin treatment (Figure 2A–C). Hematoxylin and eosin staining of the liver confirmed these data (Figure 2D). In addition, the upregulation of lipogenic (*Srebp1c*, *Fas*, *Scd1*, and *Acc1*) and inflammatory (*Tnfα*, *Il1b*, and *Emr1* (F4/80)) genes in the liver was significantly suppressed in the TX-H group relative to the HD group (Figure 2E,F). The lipogenic (FAS, SCD1, and ACC) and inflammatory (TNFα) protein expression levels were also upregulated in the HD group, and it was significantly decreased in the TX-H group relative to the HD group (Figure 2G,H). Collectively, these findings suggest that Taxifolin is capable of preventing the development of obesity and hepatic steatosis.

### 3.2. Molecular Mechanism Underlying the Anti-Obesity Effects of Taxifolin

**Figure 3:**
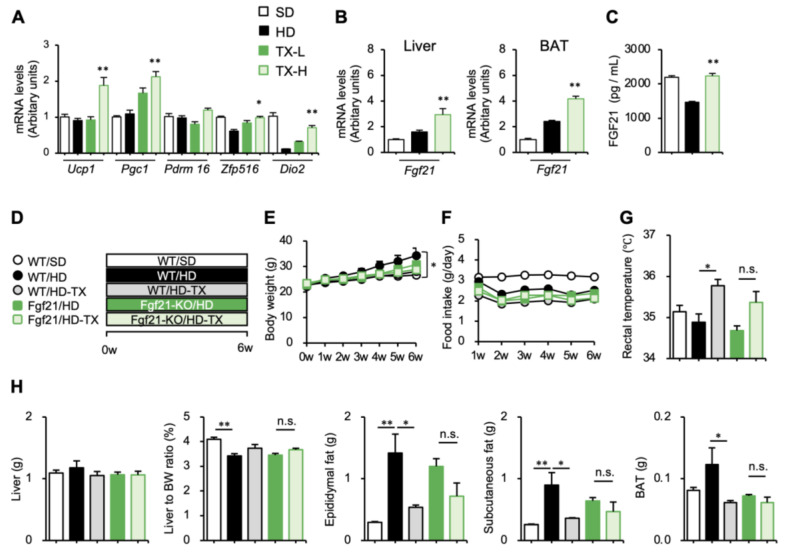
Involvement of Fgf21 in Taxifolin-mediated anti-obesity effects. (**A**–**C**) Male C57BL/6J mice were divided into the following 4 groups: white square, SD; black square, HD; dark-green square, TX-L; light-green square, TX-H. *n* = 6 in each group. (**A**) Expression levels of genes related to brown adipocyte activation (*Ucp1*, *Pgc1*, *Prdm16*, *Zfp516*, and *Dio2*) in interscapular brown adipose tissue of the C57BL/6J mice fed an HD with or without Taxifolin for 12 weeks. (**B**) Expression levels of *Fgf21* and *Il6* mRNAs in the liver. (**C**) Serum Fgf21 concentrations after 12 weeks of HD feeding with or without Taxifolin. Mean ± SEM; *n* = 6; * *p* < 0.05 and ** *p* < 0.01 vs. HD. (**D**–**H**) Male C57BL/6J mice (wild-type, WT) and Fgf21-deficient mice (Fgf21-KO) were divided into the following 5 groups: white square, WT/SD; black square, WT/HD; gray square, TX/H; dark-green square, Fgf21-KO/HD; light-green square, Fgf21-KO/HD-TX-H. *n* = 6 in each group. (**D**) Experimental protocol: *Fgf21*-deificient and wild-type mice were fed an HD with or without Taxifolin for 6 weeks; (**E**) growth curve; (**F**) food intake; (**G**) rectal temperature; (**H**) tissue weights: liver, liver-to-body weight ratio, epididymal fat, subcutaneous fat, and interscapular brown adipose tissue. Values are presented as the means; *n* = 5–6; significant differences: * *p* < 0.05 and ** *p* < 0.01.

Regarding the increased rectal temperature in the TX-H group, we found that the Taxifolin treatment increased the mRNA expression of genes related to brown adipose tissue activation such as uncoupling protein-1 (*Ucp1*) in brown adipose tissue (Figure 3A). Brown adipose tissue is involved in nonshivering thermogenesis during cold exposure and diet-induced thermogenesis, thereby contributing to whole-body energy expenditure [25]. Of note, the mRNA expression of *Fgf21*, a potent inducer of thermogenic genes in brown adipose tissue, was increased in the liver and brown adipose tissue by the Taxifolin treatment (Figure 3B). In line with this, Taxifolin treatment effectively restored the otherwise reduced serum Fgf21 concentrations in the diet-induced obese mice (Figure 3C). These observations led us to examine the involvement of Fgf21 in the Taxifolin-mediated anti-obesity effects. In this study, high-dose Taxifolin was orally administered to *Fgf21*-deficient and wild-type mice fed an HD for 6 weeks (Figure 3D). The suppressive effects of Taxifolin on body weight, adipose tissue weights, and rectal temperature were partially reduced in the Fgf21-deficient mice, whereas the treatment did not affect food intake (Figure 3E–H). These findings suggest that Taxifolin potently suppresses the development of obesity, at least partly, through Fgf21 production.

**Figure 4:**
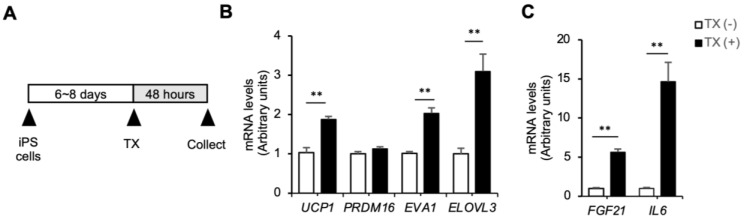
Direct action of Taxifolin on brown adipocytes: (**A**) experimental protocol: human iPS cell-derived brown adipocytes (hiPSCdBAs) were differentiated and then treated with Taxifolin at 100 μM for 48 h; (**B**) expression levels of genes related to brown adipocyte markers (*UCP1*, *PRDM16*, *EVA1*, and *ELOVL3*); (**C**) expression levels of *FGF21* and *IL6* mRNAs in the hiPSCdBAs. Values are presented as the means ± SEM; *n* = 3; significant differences: ** *p* < 0.05 vs. hiPSCdBAs without Taxifolin treatment.

Moreover, we investigated the direct effects of Taxifolin on brown adipocytes. In addition to body temperature, the expression of “BATokines”, secreted factors from mature brown adipocytes, is useful for evaluating brown adipose tissue activity [26]. In this study, human iPS cell-derived brown adipocytes (hiPSCdBAs) were treated with Taxifolin for 48 h and then subjected to mRNA expression experiments (Figure 4A). The Taxifolin treatment significantly increased the mRNA expression of *UCP1* and brown adipocyte-specific genes, such as *Epithelial V-like antigen 1* (*EVA1*) and *Elongation of very long chain fatty acid elongase 3* (*ELOVL3*) (Figure 4B). The Taxifolin treatment also significantly increased the mRNA expression of BATokines (*FGF21* and *IL6*) (Figure 4C). Collectively, these findings suggest that Taxifolin exerts its anti-obesity effects through at least two different pathways: directly acting on brown adipocytes and inducing Fgf21 expression in the liver.

### 3.3. Therapeutic Effects of Taxifolin on Hepatic Steatosis in Diet-Induced Obese Mice

**Figure 5:**
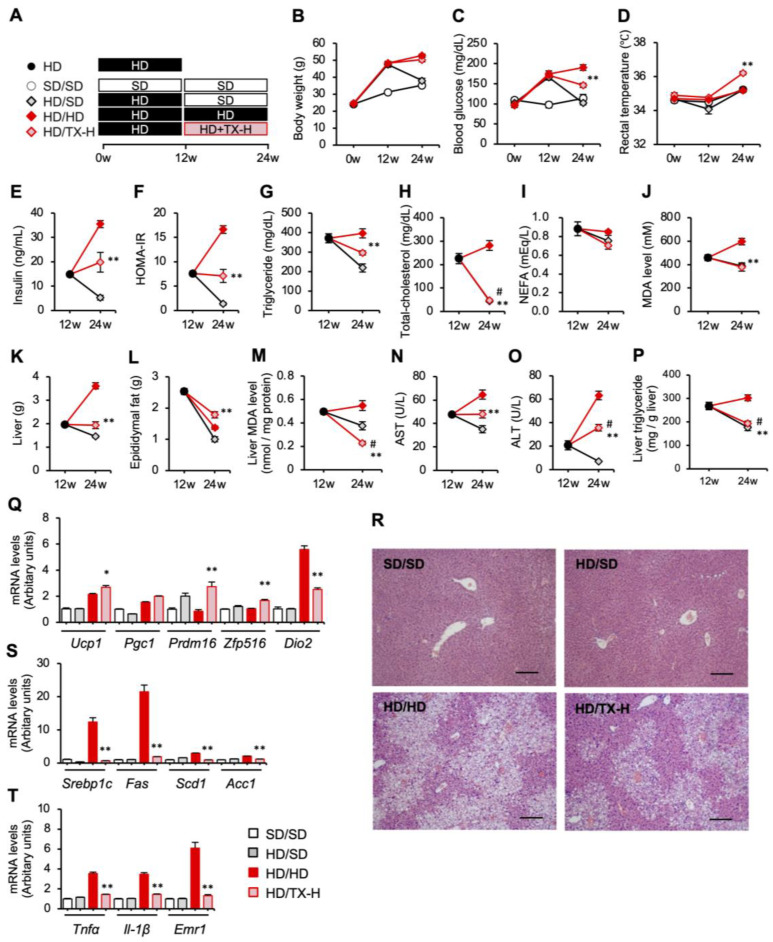
Therapeutic effects of Taxifolin on hepatic steatosis in diet-induced obese mice. (**A**) Experimental protocol: after being fed an HD for 12 weeks, C57BL/6J mice were divided into the following 3 groups and then fed the respective diets for an additional 12 weeks—HD/SD group with an SD, HD/HD group with an HD, and HD/TX-H group with an HD containing 3% (wt/wt) Taxifolin. The mice were also fed an HD for 12 weeks as the pretreatment HD group and an SD for 24 weeks as the control SD/SD group. *n* = 6 in each group. (**B**–**D**) Time course of body weight (**B**), fasting blood glucose levels (**C**), and rectal temperature (**D**). *E-P*: Metabolic parameters and tissue weights of the HD, HD/SD, HD/HD, and HD/TX-H groups. Serum concentrations of insulin (**E**), triglyceride (**G**), total cholesterol (**H**), NEFA (**I**), MDA (**J**), AST (**N**), and ALT (**O**). (**F**) HOMA-IR. Liver (**K**) and epididymal fat (**L**) tissue weights. Hepatic MDA (**M**) and triglyceride (**P**) contents. (**Q**–**T**) Four groups: white square, SD/SD; gray square, HD/SD; dark-red square, TX-L; light-red square: TX-H. (**Q**) Expression levels of genes related to brown adipocyte markers (*Ucp1*, *Pgc1*, *Prdm16*, *Zfp516*, and *Dio2*) in the interscapular brown adipose tissue. (**R**) HE staining of the liver. Scale bars: 200 µm. (**S**,**T**) Expression levels of genes related to lipogenesis (*Srebp1c*, *Fas*, *Scd1*, and *Acc1*) and inflammation (*Tnfα*, *Il1b*, and *Emr1* (F4/80)) in the liver. Values are presented as the means ± SEM; *n* = 6; significant differences: * *p* < 0.05 and ** *p* < 0.01 vs. HD/HD; # *p* < 0.05 vs. HD.

We next examined the therapeutic effects of Taxifolin after the mice developed obesity and hepatic steatosis. The mice fed an HD for 12 weeks were divided into the following three groups: HD/SD group with an SD; HD/HD group with an HD; and HD/TX-H group with an HD containing high-dose Taxifolin. Each group was then fed the respective diet for an additional 12 weeks (Figure 5A). Unlike the preventive protocol (Figure 1), Taxifolin treatment did not suppress body weight gain in the therapeutic protocol (Figure 5B). In contrast, Taxifolin was still effective for metabolic parameters, rectal temperature, hepatic steatosis, and mRNA expression in brown adipose tissue and liver (Figure 5C–T). Among others, the most striking effects were observed in the hepatic mRNA expression of genes related to lipogenesis and inflammation (Figure 5S,T).

**Figure 6:**
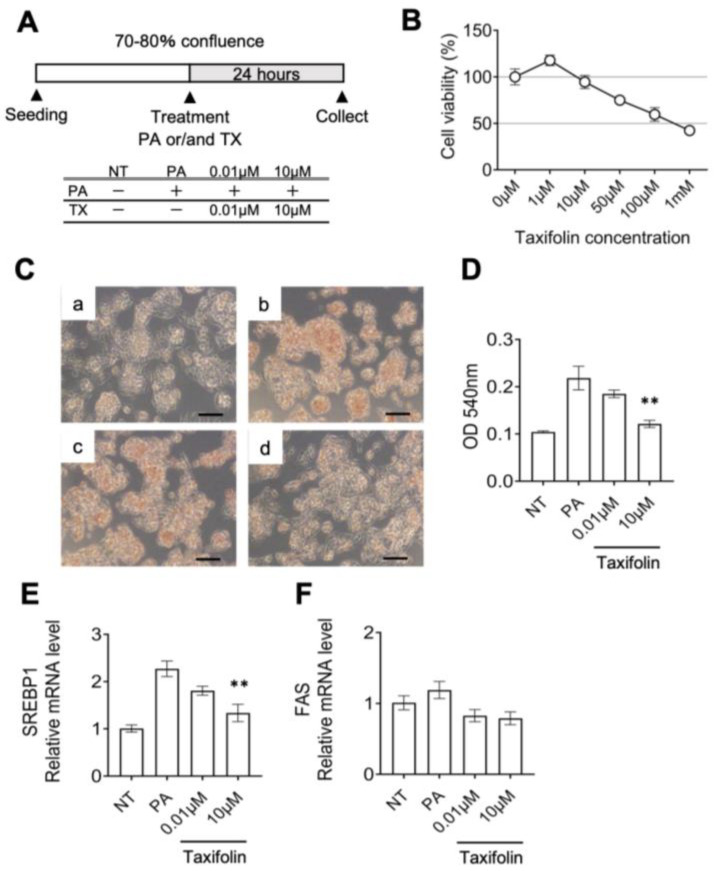
Direct action of Taxifolin on hepatocytes. (**A**) Experimental protocol: HepG2 cells were treated with Taxifolin (0.01 and 10 µM) for 24 h in the presence of palmitate (400 µM). (**B**) Cell viability after treatment with Taxifolin (1, 10, 50, and 100 µM and 1 mM) for 24 h. (**C**,**D**) Representative image of Oil Red O staining (**C**) and its quantitative evaluation measuring the absorbance at 540 nm (**D**). HepG2 cells were treated with vehicle (**a**), palmitate 400 µM (**b**), and palmitate with 0.01 µM (**c**) or 10 µM (**d**) Taxifolin for 24 h. Scale bars: 100 µm. *E* and *F*: Expression levels of *SREBP1* (**E**) and *FAS* (**F**) mRNAs in the HepG2 cells. Values are presented as the means ± SEM; *n* = 3; significant differences: ** *p* < 0.01 vs. palmitate.

We next investigated the direct effects of Taxifolin on hepatocytes using human HepG2 cells. After confirming the cell viability treated with less than 50 µM Taxifolin, HepG2 cells were treated with Taxifolin for 24 h in the presence of palmitate (Figure 6A,B). Oil Red O staining revealed that palmitate-induced lipid accumulation was suppressed by Taxifolin in a dose-dependent manner (Figure 6C,D). Taxifolin also effectively suppressed the otherwise increased mRNA expression of lipogenic genes in this experimental setting (Figure 6E,F). Collectively, these in vivo and in vitro data strongly suggest that Taxifolin can directly act on hepatocytes to ameliorate hepatic steatosis, in addition to its effects on systemic energy expenditure.

### 3.4. Preventive Effects of Taxifolin on the Development of NASH in a Murine Model

**Figure 7:**
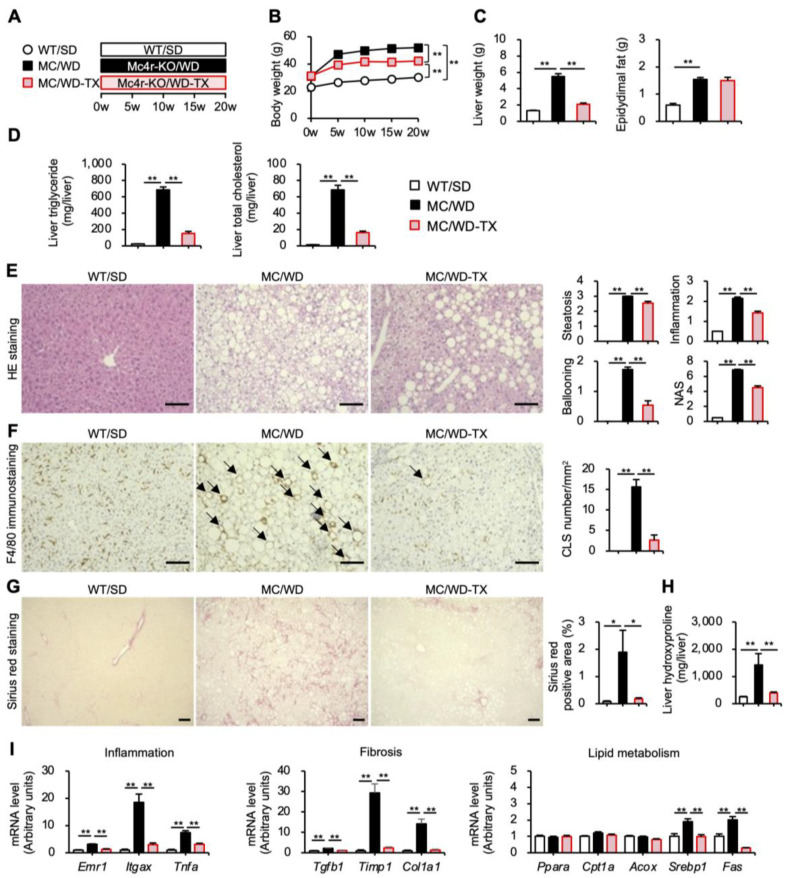
Preventive effects of Taxifolin on the development of NASH in a mouse model. (**A**) Experimental protocol: genetically obese melanocotin-4 receptor (*Mc4r*)-deficient mice on a WD with or without 3% Taxifolin for 20 weeks (MC/WD or MC/WD-TX, respectively). Wild-type mice on a standard diet for 20 weeks (WT/SD) were used as a control. (**B**) Growth curve: *C-I* 3 groups–white square, WT/SD; black square, MC/WD; light-red square, MC/WD-TX. (**C**) Liver and epididymal fat weights. (**D**) Hepatic triglyceride and total cholesterol contents. (**E**) HE staining of the liver. Histological analysis using the nonalcoholic fatty liver disease (NAFLD) activity score (NAS) system. (**F**) F4/80 immunostaining. The arrows indicate the crown-like structures (CLS). (**G**) Sirius red staining. (**H**) Hydroxyproline contents of the liver. (**I**) Expression levels of genes related to inflammation (*Emr1*, *Itgax*, and *Tnfα*), fibrosis (*Tgfb1*, *Timp1*, and *Col1a1*), and lipid metabolism (*Ppara*, *Cpt1a*, *Acox*, *Srebp1*, and *Fas*). Scale bars: 100 µm. Values are presented as the means ± SEM; *n* = 11–12; significant differences: * *p* < 0.05 and ** *p* < 0.01.

Next, we investigated the effects of Taxifolin on the development of NASH in a murine model. As a preventive protocol, genetically obese *Mc4r*-deficient mice were on a WD with or without high-dose Taxifolin for 20 weeks (Figure 7A). Consistently with the prior experiments shown in Figure 1, the Taxifolin treatment significantly suppressed the increase in body weight, liver weight, and hepatic lipid contents in a NASH model (Figure 7B–D). The Taxifolin treatment also decreased the serum concentrations of ALT, AST, and total cholesterol, whereas the serum triglyceride and blood glucose levels were not affected in this model (Supplementary Materials Table S3). After 20 weeks of WD feeding, the *Mc4r*-deficient mice showed histological features similar to human NASH, including micro–macro vesicular steatosis, ballooning degeneration (indicating hepatocyte damages), and massive infiltration of inflammatory cells (Figure 7E), as previously described [5]. The histological evaluation using the NAFLD Activity Score (NAS) system revealed that the Taxifolin treatment significantly reduced the scores of steatosis, inflammation, and ballooning degeneration (Figure 7E), suggesting the preventive effects of Taxifolin on the development of NASH. Previously, we found a unique histological structure termed CLS, where macrophages aggregate around dead hepatocytes with large lipid droplets and engulf the dead cells and residual lipids [27]. We also provided evidence that CLS is a driver that promotes liver fibrosis during the development of NASH [28]. In this study, the histological analysis revealed CLS formation and liver fibrosis (pericellular fibrosis) in *Mc4r*-deficient mice fed a WD, which was markedly suppressed by the Taxifolin treatment (Figure 7F,G). The measurement of hydroxyproline contents of the liver confirmed the data on liver fibrosis (Figure 7H). Consistently, the Taxifolin treatment inhibited the upregulation of the mRNA levels related to inflammation, fibrosis, and lipid metabolism in this NASH model (Figure 7I). In particular, *Itgax* (Cd11c) was selectively expressed in the macrophages within the CLS, which possess profibrotic properties [28]. Taken together, these results indicate that Taxifolin can prevent the development of hepatic steatosis and subsequent liver fibrosis in a NASH model.

### 3.5. Therapeutic Effects of Taxifolin during the Progression from NASH to Liver Tumors

**Figure 8:**
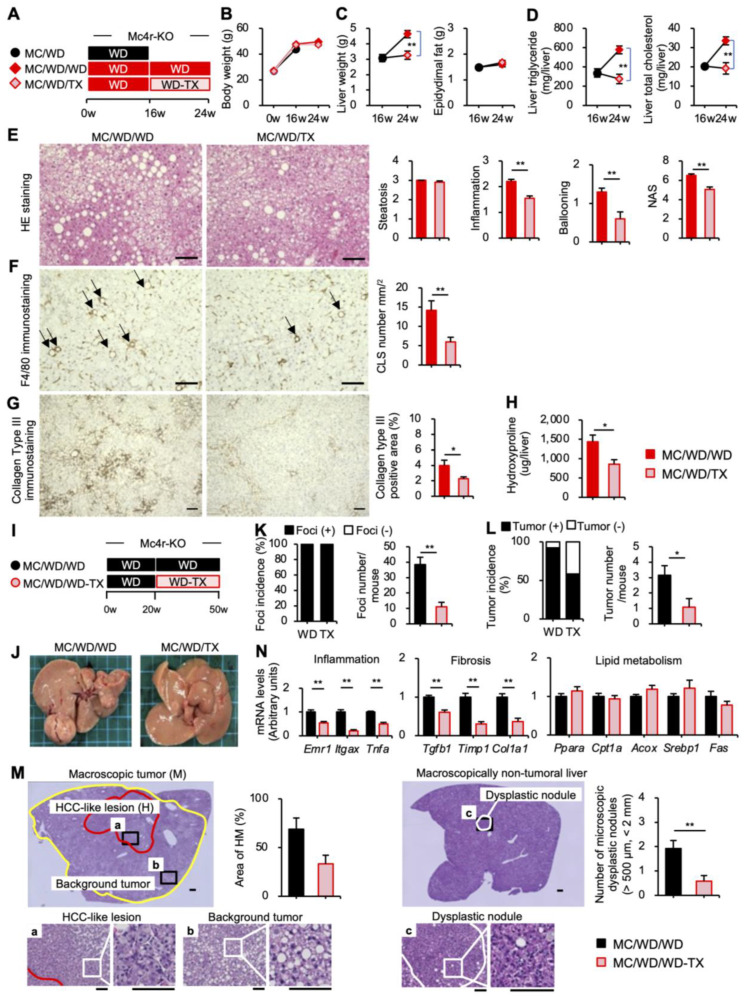
Therapeutic effects of Taxifolin on the progression of NASH in a mouse model. (**A**) Experimental protocol: the *Mc4r*-deficient mice were on a WD for 16 weeks to develop NASH and then treated with or without 3% Taxifolin for an additional 8 weeks (MC/WD/WD or MC/WD/TX, respectively). (**B**) Growth curve; (**C**) liver and epididymal fat weights; (**D**) hepatic triglyceride and total cholesterol contents. (**E**–**H**) Two groups: dark-red square, MC/WD/WD; light-red square, MC/WD/WD-TX. (**E**) HE staining of the liver. Histological analysis using the NAS. Scale bars: 100 µm. (**F**) F4/80 immunostaining of the liver. The arrows indicate the CLS. (**G**) Immunostaining for collagen type III of the liver. (**H**) Hydroxyproline contents of the liver. Scale bars: 100 µm; *n* = 10–11; * *p* < 0.05 and ** *p* < 0.01. (**I**) experimental protocol: the *Mc4r*-deficient mice were fed a WD for 20 weeks to develop NASH and then treated with or without 3% Taxifolin for an additional 30 weeks (MC/WD/WD or MC/WD/TX, respectively). (**J**) Representative image of the gross appearance of the livers. (**K**–**M**) Two groups: black square, MC/WD/WD; light-red square, MC/WD/WD-TX. (**K**,**L**) Incidence and multiplicity of foci (**K**) and tumors (**L**) in the liver. (**M**) Representative images of the HE staining of the macroscopic tumoral (left) and nontumoral (right) lesions. The areas defined by yellow and red lines indicate a grossly detectable tumor and an HCC-like lesion that can only be detected by histological examination, respectively. a–c: A higher magnification view of the HCC-like lesion (a), background tumor (b), and dysplastic nodule (c). (**N**): Expression levels of genes related to inflammation (*Emr1*, *Itgax*, and *Tnfα*), fibrosis (*Tgfb1*, *Timp1*, and *Col1a1*), and lipid metabolism (*Pparα*, *Cpt1a*, *Acox*, *Srebp1*, and *Fas*) in nontumorous lesions of the liver. Values are presented as the means ± SEM; *n* = 13 and 12, for MC/WD/WD and MC/WD/TX, respectively; significant differences: ** *p* < 0.01.

We next assessed whether Taxifolin has a therapeutic potential for NASH. The *Mc4r*-deficient mice were on a WD for 16 weeks to develop NASH-like liver phenotypes, and the mice were then further fed a WD with or without Taxifolin for an additional 8 weeks (Figure 8A). Similar to the preventive protocol (Figure 7), the Taxifolin treatment significantly suppressed the liver weight and hepatic lipid contents, whereas the treatment did not affect body weight gain in the therapeutic protocol (Figure 8B–D). The Taxifolin treatment also reduced the serum concentrations of ALT and AST, whereas the serum concentrations of total cholesterol and triglyceride and blood glucose levels were not affected in this model (Supplementary Materials Table S4). The histological examinations revealed that the Taxifolin treatment significantly ameliorated hepatic steatosis, CLS formation, and liver fibrosis (Figure 8E–H). The data were confirmed by mRNA expression (Supplementary Materials Figure S2).

Finally, we investigated whether Taxifolin can prevent the progression from NASH to liver tumors. The *Mc4r*-deficient mice were on a WD for 20 weeks to develop NASH, and then the mice were further fed a WD with or without Taxifolin for an additional 30 weeks (Figure 8I). As we previously reported [3], these mice developed multiple liver tumors, which could also be detected by macroscopic observations of the surface of the liver (Figure 8J). We grossly examined the number of foci and tumors according to their size and found that the Taxifolin treatment markedly suppressed the number of foci and tumors (Figure 8K,L, Supplementary Materials Figure S1). Interestingly, close histological examination of the tissue sections obtained from the grossly observed tumors revealed that they were histologically heterogeneous, with some areas resembling human HCC and others being composed of proliferative dysplastic hepatocytes with steatosis (Figure 8M, left). In the HCC-like lesions, the tumor cells exhibited a uniform morphology with enlarged and hyperchromatic nuclei, and they formed irregular and thick trabeculae consisting of two or more cells, accompanied by the loss of normal liver architecture (Figure 8Ma,b). Although not reaching a statistically significant difference, the Taxifolin treatment tended to decrease the area of HCC-like lesions (Figure 8M, left). Furthermore, our histological observations also detected small dysplastic nodules with diameters between 0.5 and 2 mm in the macroscopically nontumoral NASH liver, where we found proliferation of atypical hepatocytes with enlarged and hyperchromatic nuclei (Figure 8M, right). There were fewer microscopic dysplastic nodules in the Taxifolin treatment group than the control groups (Figure 8M, right). Finally, we found that the Taxifolin treatment significantly reduced the mRNA expression of genes related to inflammation and fibrosis in the tumorous lesions of the liver, without affecting *Cd206*, a representative marker for tumor-associated macrophages (Supplementary Materials Figure S3). Taken together, these observations suggest that Taxifolin potently prevents the progression from NASH to liver tumors in a murine model.

## 4. Discussion

In this study, we demonstrated that the Taxifolin treatment markedly prevents the development of hepatic steatosis, NASH, and liver tumors in a mouse model. In particular, Taxifolin is effective on lipid accumulation, chronic inflammation, and fibrosis in the liver when the treatment starts after the mice develop hepatic steatosis and NASH. Although the precise mechanisms of action of Taxifolin remain to be fully elucidated, our data suggest that Taxifolin directly acts on hepatocytes and brown adipocytes to suppress lipogenesis and activate energy expenditure, respectively. Moreover, we found that the Taxifolin treatment effectively prevents the progression from NASH to liver tumors. To date, numerous clinical studies have been conducted for NASH in which the primary propositions include the resolution of NASH without the worsening of fibrosis or the improvement of fibrosis without the resolution of NASH [29]. Considering the long-term outcome, there is a need to investigate the effects of novel medicines on liver tumorigenesis. However, it is technically difficult because of a lack of appropriate animal models that develop hepatic steatosis, NASH, and liver tumors, sequentially. In this regard, using our unique animal model, we provided evidence of Taxifolin’s therapeutic potential in hepatic steatosis, NASH, and liver tumors, with high safety and long-term efficacy, because it is already widely used as a health supplement.

In this study, we confirmed the anti-obese and antidiabetic effects of Taxifolin using two different mouse models (i.e., diet-induced and genetically obese mice). For its underlying mechanism, we found that Taxifolin increases the activity of brown adipose tissue at least through two distinct pathways: direct action on brown adipocytes and indirect action via FGF21 production. Since there are species differences in the cellular functions and markers of brown adipocytes, we employed human iPS cell-derived brown adipocytes to examine the direct effects of Taxifolin. In addition to the genes related to differentiation and thermogenesis, BATokines are supposed to play a key role in energy homeostasis. Indeed, while the contribution of brown adipose tissue to whole-body energy expenditure is not evident in humans, accumulating evidence supports the significant role of brown adipose tissue in ameliorating systemic metabolic conditions [30,31,32,33]. In contrast, the activation of brown adipose tissue may be involved in cancer cachexia [34,35]. In this regard, our data show that Taxifolin prevents tumorigenesis in the liver without inducing cachexia, suggesting the appropriate brown adipose tissue activation. Collectively, this study provides novel insight into the clinical translation of Taxifolin for the treatment of obesity and its complications.

It is important to discuss the potential mechanisms of Taxifolin-mediated antitumor effects in a mouse model of NASH. Accumulating evidence indicates that tumor-associated macrophages with anti-inflammatory properties enhance tumor growth and induce resistance against conventional antitumor therapies. On the other hand, sustained low-grade inflammation has been implicated in the pathogenesis of liver fibrosis, which plays a pivotal role in carcinogenesis. In this study, Taxifolin effectively suppressed hepatic steatosis, inflammation, and fibrosis in our NASH model. Similar anti-inflammatory and antifibrotic effects were observed in tumorous lesions of the liver. Therefore, it is conceivable that Taxifolin suppresses tumor development of the liver, mainly through inhibiting chronic inflammation in nontumorous lesions. In line with this, several studies reported that Taxifolin inhibits pro-inflammatory cytokine expression and NF-κB activation in cultured macrophages [36,37]. Further studies are required to evaluate the direct effect of Taxifolin on the growth of tumor cells.

As the body-weight-lowering effects of Taxifolin were relatively mild when Taxifolin was administered to obese mice, it is important to determine how Taxifolin regulates NASH-like liver phenotypes. Indeed, the Taxifolin treatment markedly suppressed hepatic expression of genes related to lipogenesis, suggesting a direct action of Taxifolin on hepatocytes. Consistently, Taxifolin significantly inhibited the palmitate-induced lipid accumulation and upregulation of lipogenic genes in cultured hepatocytes. Of note, based on previous studies [38], the dose of Taxifolin used in our in vitro study is considered to be within the physiological range, although we did not determine its serum concentrations in our mouse models. Taxifolin is also known to suppress proinflammatory cytokine expression in cultured macrophages in vitro and chemically induced liver fibrosis in vivo [18,39]. Several studies suggest the involvement of Nrf-2 (nuclear factor erythroid 2-related factor 2), HO-1 (heme oxygenase 1), and AMPK (AMP-activated protein kinase) as the underlying mechanism of the antioxidative properties of Taxifolin [8,40,41]. Given that the NASH pathogenesis is heterogenous and diverse in humans, these pleiotropic effects of Taxifolin may be advantageous for clinical practice. In this respect, recent studies developed a novel nanocomplex of selenium with sorafenib [42] or Taxifolin [43], which exhibits more beneficial effects than those of single molecules in vitro in terms of anticancer or neuroprotective effects. Accordingly, the use of the nanocomplex would be a valuable therapeutic option for NASH. Our next step will be to explore the carcinogenic mechanism of Taxifolin on NASH using different animal models, although there are few animal models suitable for investigating the NASH continuum.

## 5. Conclusions

In summary, we demonstrated the novel therapeutic potentials of Taxifolin, a unique bioactive flavonoid, for obesity-induced hepatic steatosis, fibrogenesis, and tumorigenesis in mice. Previous studies have pointed to the anti-obesity, antidiabetic, anti-inflammatory, and antitumor effects of Taxifolin in various in vitro and in vivo models [8]. In addition, this study provides evidence that Taxifolin is effective on the NASH continuum. Our data also provide insight into the novel mechanisms of action of Taxifolin and collectively may pave the way for the clinical translation of Taxifolin.

## Data Availability

The data that support the findings of this study are available from the corresponding author upon reasonable request.

## Supplementary Materials

The following supporting information can be downloaded at: https://www.mdpi.com/article/10.3390/nu15020350/s1, Figure S1: Representative image of the gross appearance of livers; Figure S2: Expression levels of genes related to inflammation, fibrosis, and lipid metabolism in the liver of a NASH model treated with Taxifolin; Figure S3: Expression levels of genes related to inflammation, fibrosis, and tumor-associated macrophages in tumorous lesions of the liver in a mouse model of NASH treated with Taxifolin; Table S1: Primers for mice used in the present study; Table S2: Primers for humans used in the present study; Table S3: Preventive effects of Taxifolin on serum parameters in *Mc4r*-deficient mice fed a Western diet; Table S4: Therapeutic effects of Taxifolin on serum parameters in *Mc4r*-deficient mice fed a Western diet after the mice developed NASH.
