# Supplementary material for: Novel Therapeutic Potentials of Taxifolin for Obesity-Induced Hepatic Steatosis, Fibrogenesis, and Tumorigenesis
Source: Nutrients. 2023 Jan 10;15(2):350. doi: 10.3390/nu15020350 (PMC9865844; doi:10.3390/nu15020350)
Supplement: Supplementary file 1 [file nutrients-15-00350-s001.zip › nutrients-2093052 supplementary.pdf]

Supplementary Materials

MC/WD/WD MC/WD/TX

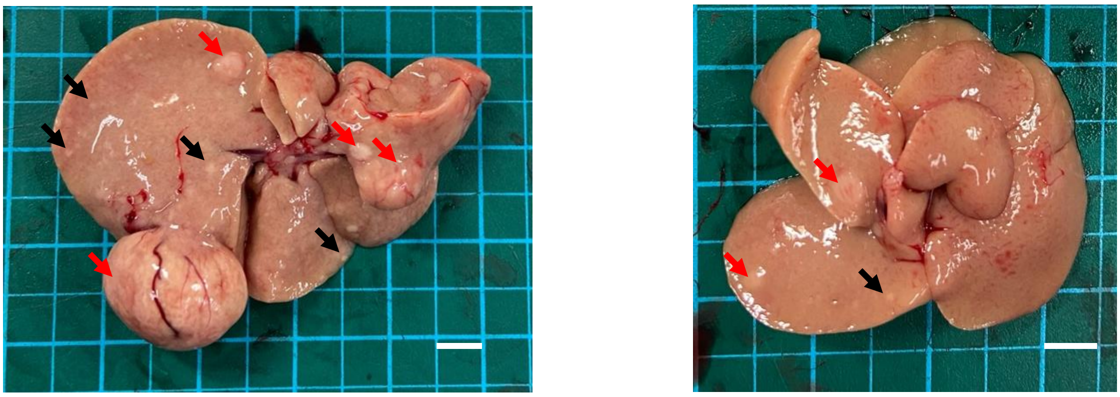

Figure S1. Representative image of gross appearance of livers.

For assessment of tumor development, the number and the size of lumps were measured in the liver, in which the lumps less than 1 mm and larger than 1 mm were considered as foci (black arrows) and tumors (red arrows), respectively. Scale bars: 5mm.

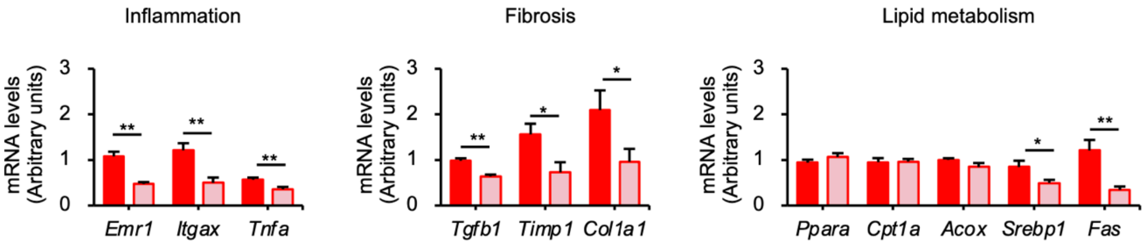

Figure S2. Expression levels of genes related to inflammation, fibrosis, and lipid metabolism in the liver of a NASH model treated with Taxifolin.

Mc4r-deficient mice were fed a Western diet for 16 weeks to develop NASH, and then treated with or without 3% of Taxifolin for additional 8 weeks (MC/WD/WD or MC/WD/TX, respectively). Hepatic expression levels of genes related to inflammation, fibrosis, and lipid metabolism were measured. $n = 10–11$, $*P < 0.05$, $**P < 0.01$.

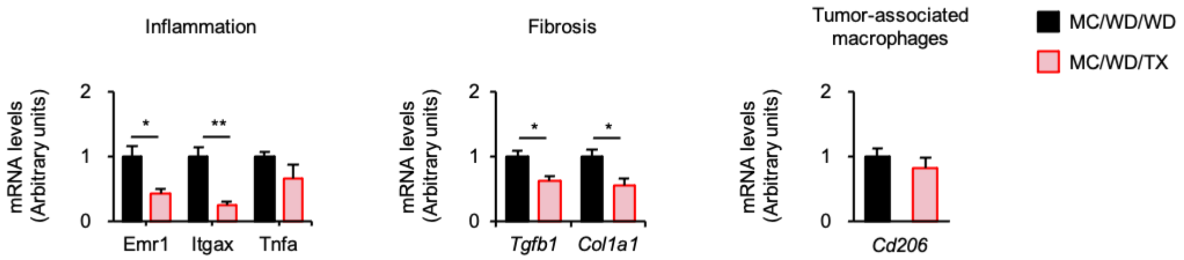

Figure S3. Expression levels of genes related to inflammation, fibrosis, and tumor associated macrophage in tumorous lesions of the liver in a mouse model of NASH treated with Taxifolin.

Mc4r-deficient mice were fed a Western Diet for 20 weeks to develop NASH, and then treated with or without 3% of Taxifolin for additional 30 weeks (MC/WD/WD or MC/WD/TX, respectively). Hepatic expression levels of genes related to inflammation, fibrosis, and tumor-associated macrophages (*Cd206*) were measured. n = 7-12, * $P < 0.05$, ** $P < 0.01$.

Table S1. Primers for mice used in the present study

Genes	Primers	Primers
Acc1	Forward	TGAGATTGGCATGGTAGCCTG
	Reverse	CTCGGCCATCTGGATATTCAG
Acox	Forward	GCCTTTGTTGTCCCTATCCGT
	Reverse	CGATATCCCCAACAGTGATGC
Col1a1	Forward	CCTCAGGGTATTGCTGGACAAC
	Reverse	ACCACTTGATCCAGAAGGACCTT
Cpt1a	Forward	CCTGCATTCCTTCCCATTTG
	Reverse	TGCCCATGTCCTTGTAATGTG
Dio2	Forward	CAGTGTGGTGCACGTCTCCAATC
	Reverse	TGAACCAAAGTTGACCACCAG
Emr1	Forward	CTTTGGCTATGGGCTTCCAGTC
	Reverse	GCAAGGAGGACAGAGTTTATCGTG
Fasn	Forward	CCTGGATAGCATTCCGAACCT
	Reverse	AGCACATCTCGAAGGCTACACA
Il-1b	Forward	CTGAACTCAACTGTGAAATGCCA
	Reverse	AAAGGTTTGGAAGCAGCCCT
Itgax	Forward	GCCATTGAGGGCACAGAGA
	Reverse	GAAGCCCTCCTGGGACATCT
Pgc1a	Forward	CCCTGCCATTGTTAAGACC
	Reverse	TGCTGCTGTTCCTGTTTTC
Ppara	Forward	AGGAAGCCGTTCTGTGACAT
	Reverse	AATCCCCTCCTGCAACTTCT
Prdm16	Forward	CAGCACGGTGAAGCCATTC
	Reverse	GCGTGCATCCGCTTGTG
Scd1	Forward	CATCATTCTCATGGTCCTGCT
	Reverse	CCCAGTCGTACACGTCATTTT
Srebp1	Forward	GGAGCCATGGATTGCACATT
	Reverse	GGCCCGGGAAGTCACTGT
Tgfb1	Forward	CCTGAGTGGCTGTCTTTTGACG
	Reverse	AGTGAGCGCTGAATCGAAAGC
Timp1	Forward	CATCACGGGCCGCCTA
	Reverse	AAGCTGCAGGCACTGATGTG
Tnfa	Forward	ACCCTCACACTCAGATCATCTTC
	Reverse	TGGTGGTTTGCTACGACGT
Ucp1	Forward	TACACGGGGACCTACAATGCT

	Reverse	TCGCACAGCTTGGTACGCTT
18s	Forward	CGATGCTCTTAGCTGAGTGT
	Reverse	GGTCCAAGAATTTCACCTCT
36B4	Forward	GGCCCTGCACTCTCGCTTTC
	Reverse	TGCCAGGACGCGCTTGT

Table S2. Primers for humans used in the present study

Genes	Primers	Primers
EVA1	Forward	GGAATCCTGAGCGGTACGATG
	Reverse	ATCCTGCCTCCACATGTACG
ELOVL3	Forward	CACTGGTACCACCACAGCAC
	Reverse	ATCCTGCCTCCACATGTACG
FASN	Forward	AACCGGCTCTCCTTCTTCTTCGACTT
	Reverse	TCCGAGCGGCAGTACCCATTC
GAPDH	Forward	CCACTCCTCCACCTTTGAC
	Reverse	ACCCTGTTGCTGTAGCCA
Prdm16	Forward	CGAGGCCCCTGTCTACATTC
	Reverse	GCTCCCATCCGAAGTCTGTC
Srebp1	Forward	AAACTCAAGCAGGAGAACCTAAGTCT
	Reverse	GTCAGTGTGTCCTCCACCTCAGT
Ucp1	Forward	AAATCAGCTCCGCCTCTCTC
	Reverse	TGCCACTCCTCCAGTCGTTA
18S	Forward	CCTGGATACCGCAGCTAGGA
	Reverse	GCGGCGCAATACGAATGCCCC

Table S3. Preventive effects of Taxifolin on serum parameters in *Mc4r*-deficient mice fed a Western diet

Variable	WT/SD	MC/WD	MC/WD-TX
ALT (U/L)	29.2 ± 2.5**	355.5 ± 28.7	186.1 ± 35.8**
AST (U/L)	104.8 ± 9.6**	343.1 ± 44.9	157.6 ± 17.0**
TC (mg/dL)	69.2 ± 2.0**	288.1 ± 14.2	178.7 ± 5.7**
TG (mg/dL)	69.7 ± 4.8	57.0 ± 5.4	57.8 ± 3.5
BG-5w (mg/dL)	156.8 ± 6.2	159.2 ± 8.4	173.1 ± 8.7
BG-10w (mg/dL)	149.0 ± 5.3	173.1 ± 9.6	173.9 ± 6.6
BG-19w (mg/dL)	162.3 ± 6.4	142.3 ± 4.2	164.3 ± 9.0

ALT: alanine aminotransferase, AST: aspartate aminotransferase, TC: total cholesterol, TG: triglyceride, BG: blood glucose. Data are expressed as mean ± SE. *$P < 0.05$, **$P < 0.01$ versus MC/WD

Table S4. Therapeutic effects of Taxifolin on serum parameters in *Mc4r*-deficient mice fed a Western diet after the mice developed NASH.

Variable	MC/WD	MC/WD/WD	MC/WD/TX
ALT (U/L)	305.4 ± 79.6	373.7 ± 70.0	193.8 ± 20.5
AST (U/L)	202.5 ± 24.3*	306.3 ± 32.9	176.5 ± 18.7**
TC (mg/dL)	209.5 ± 6.8**	268.6 ± 14.1	237.5 ± 13.9
TG (mg/dL)	53.1 ± 2.6	81.3 ± 15.0	83.5 ± 12.6
BG (mg/dL)	187.8 ± 13.2	199.8 ± 6.5	190.4 ± 10.7

ALT: alanine aminotransferase, AST: aspartate aminotransferase, TC: total cholesterol, TG: triglyceride, BG: blood glucose. Data are expressed as mean ± SE. *$P < 0.05$, **$P < 0.01$ versus MC/WD/WD
